# Fine-Mapping of the 1p11.2 Breast Cancer Susceptibility Locus

**DOI:** 10.1371/journal.pone.0160316

**Published:** 2016-08-24

**Authors:** Hisani N. Horne, Charles C. Chung, Han Zhang, Kai Yu, Ludmila Prokunina-Olsson, Kyriaki Michailidou, Manjeet K. Bolla, Qin Wang, Joe Dennis, John L. Hopper, Melissa C. Southey, Marjanka K. Schmidt, Annegien Broeks, Kenneth Muir, Artitaya Lophatananon, Peter A. Fasching, Matthias W. Beckmann, Olivia Fletcher, Nichola Johnson, Elinor J. Sawyer, Ian Tomlinson, Barbara Burwinkel, Frederik Marme, Pascal Guénel, Thérèse Truong, Stig E. Bojesen, Henrik Flyger, Javier Benitez, Anna González-Neira, Hoda Anton-Culver, Susan L. Neuhausen, Hermann Brenner, Volker Arndt, Alfons Meindl, Rita K. Schmutzler, Hiltrud Brauch, Ute Hamann, Heli Nevanlinna, Sofia Khan, Keitaro Matsuo, Hiroji Iwata, Thilo Dörk, Natalia V. Bogdanova, Annika Lindblom, Sara Margolin, Arto Mannermaa, Veli-Matti Kosma, Georgia Chenevix-Trench, kConFab/AOCS Investigators, Anna H. Wu, David ven den Berg, Ann Smeets, Hui Zhao, Jenny Chang-Claude, Anja Rudolph, Paolo Radice, Monica Barile, Fergus J. Couch, Celine Vachon, Graham G. Giles, Roger L. Milne, Christopher A. Haiman, Loic Le Marchand, Mark S. Goldberg, Soo H. Teo, Nur A. M. Taib, Vessela Kristensen, Anne-Lise Borresen-Dale, Wei Zheng, Martha Shrubsole, Robert Winqvist, Arja Jukkola-Vuorinen, Irene L. Andrulis, Julia A. Knight, Peter Devilee, Caroline Seynaeve, Montserrat García-Closas, Kamila Czene, Hatef Darabi, Antoinette Hollestelle, John W. M. Martens, Jingmei Li, Wei Lu, Xiao-Ou Shu, Angela Cox, Simon S. Cross, William Blot, Qiuyin Cai, Mitul Shah, Craig Luccarini, Caroline Baynes, Patricia Harrington, Daehee Kang, Ji-Yeob Choi, Mikael Hartman, Kee Seng Chia, Maria Kabisch, Diana Torres, Anna Jakubowska, Jan Lubinski, Suleeporn Sangrajrang, Paul Brennan, Susan Slager, Drakoulis Yannoukakos, Chen-Yang Shen, Ming-Feng Hou, Anthony Swerdlow, Nick Orr, Jacques Simard, Per Hall, Paul D. P. Pharoah, Douglas F. Easton, Stephen J. Chanock, Alison M. Dunning, Jonine D. Figueroa

**Affiliations:** 1 Division of Cancer Epidemiology and Genetics, National Cancer Institute, Rockville, MD, United States of America; 2 Food and Drug Administration, Silver Spring, MD, United States of America; 3 Centre for Cancer Genetic Epidemiology, Department of Public Health and Primary Care, University of Cambridge, Cambridge, UK; 4 Centre for Epidemiology and Biostatistics, School of Population and Global Health, The University of Melbourne, Melbourne, Australia; 5 Department of Pathology, The University of Melbourne, Melbourne, Australia; 6 Netherlands Cancer Institute, Antoni van Leeuwenhoek hospital, Amsterdam, The Netherlands; 7 Division of Health Sciences, Warwick Medical School, Warwick University, Coventry, UK; 8 Institute of Population Health, University of Manchester, Manchester, UK; 9 Department of Gynaecology and Obstetrics, University Hospital Erlangen, Friedrich-Alexander University Erlangen-Nuremberg, Comprehensive Cancer Center Erlangen-EMN, Erlangen, Germany; 10 David Geffen School of Medicine, Department of Medicine Division of Hematology and Oncology, University of California at Los Angeles, Los Angeles, CA, United States of America; 11 Breakthrough Breast Cancer Research Centre, The Institute of Cancer Research, London, UK; 12 Division of Breast Cancer Research, The Institute of Cancer Research, London, UK; 13 Research Oncology, Guy’s Hospital, King's College London, London, UK; 14 Wellcome Trust Centre for Human Genetics and Oxford Biomedical Research Centre, University of Oxford, Oxford, UK; 15 Department of Obstetrics and Gynecology, University of Heidelberg, Heidelberg, Germany; 16 Molecular Epidemiology Group, German Cancer Research Center (DKFZ), Heidelberg, Germany; 17 National Center for Tumor Diseases, University of Heidelberg, Heidelberg, Germany; 18 Environmental Epidemiology of Cancer, Center for Research in Epidemiology and Population Health, INSERM, Villejuif, France; 19 University Paris-Sud, Villejuif, France; 20 Copenhagen General Population Study, Herlev Hospital, Copenhagen University Hospital, Herlev, Denmark; 21 Department of Clinical Biochemistry, Herlev Hospital, Copenhagen University Hospital, Herlev, Denmark; 22 Faculty of Health and Medical Sciences, University of Copenhagen, Copenhagen, Denmark; 23 Department of Breast Surgery, Herlev Hospital, Copenhagen University Hospital, Herlev, Denmark; 24 Human Cancer Genetics Program, Spanish National Cancer Research Centre, Madrid, Spain; 25 Centro de Investigación en Red de Enfermedades Raras, Valencia, Spain; 26 Department of Epidemiology, University of California Irvine, Irvine, CA, United States of America; 27 Beckman Research Institute of City of Hope, Duarte, CA, United States of America; 28 Division of Clinical Epidemiology and Aging Research, German Cancer Research Center (DKFZ), Heidelberg, Germany; 29 Division of Preventive Oncology, German Cancer Research Center (DKFZ), Heidelberg, Germany; 30 German Cancer Consortium (DKTK), German Cancer Research Center (DKFZ), Heidelberg, Germany; 31 Division of Gynaecology and Obstetrics, Technische Universität München, Munich, Germany; 32 Division of Molecular Gyneco-Oncology, Department of Gynaecology and Obstetrics, University Hospital of Cologne, Cologne, Germany; 33 Center of Familial Breast and Ovarian Cancer, University Hospital of Cologne, Cologne, Germany; 34 Center for Integrated Oncology, University Hospital of Cologne, Cologne, Germany; 35 Center for Molecular Medicine, University Hospital of Cologne, Cologne, Germany; 36 Dr. Margarete Fischer-Bosch-Institute of Clinical Pharmacology, Stuttgart, Germany; 37 University of Tübingen, Tübingen, Germany; 38 Molecular Genetics of Breast Cancer, German Cancer Research Center (DKFZ), Heidelberg, Germany; 39 Department of Obstetrics and Gynecology, Helsinki University Hospital, University of Helsinki, Helsinki, Finland; 40 Department of Preventive Medicine, Kyushu University Faculty of Medical Sciences, Fukuoka, Japan; 41 Department of Breast Oncology, Aichi Cancer Center Hospital, Aichi, Japan; 42 Gynaecology Research Unit, Hannover Medical School, Hannover, Germany; 43 Department of Radiation Oncology, Hannover Medical School, Hannover, Germany; 44 Department of Molecular Medicine and Surgery, Karolinska Institutet, Stockholm, Sweden; 45 Department of Oncology - Pathology, Karolinska Institutet, Stockholm, Sweden; 46 Cancer Center, Kuopio University Hospital, Kuopio, Finland; 47 Institute of Clinical Medicine, Pathology and Forensic Medicine, University of Eastern Finland, Kuopio, Finland; 48 Imaging Center, Department of Clinical Pathology, Kuopio University Hospital, Kuopio, Finland; 49 Department of Genetics, QIMR Berghofer Medical Research Institute, Brisbane, Australia; 50 Peter MacCallum Cancer Center, The University of Melbourne, Melbourne, Australia; 51 Department of Preventive Medicine, Keck School of Medicine, University of Southern California, Los Angeles, CA, United States of America; 52 University Hospital Gashuisberg, Leuven, Belgium; 53 Vesalius Research Center, Leuven, Belgium; 54 Laboratory for Translational Genetics, Department of Oncology, University of Leuven, Leuven, Belgium; 55 Division of Cancer Epidemiology, German Cancer Research Center (DKFZ), Heidelberg, Germany; 56 University Cancer Center Hamburg (UCCH), University Medical Center Hamburg-Eppendorf, Hamburg, Germany; 57 Unit of Molecular Bases of Genetic Risk and Genetic Testing, Department of Preventive and Predictive Medicine, Fondazione IRCCS (Istituto Di Ricovero e Cura a Carattere Scientifico) Istituto Nazionale dei Tumori (INT), Milan, Italy; 58 Division of Cancer Prevention and Genetics, Istituto Europeo di Oncologia, Milan, Italy; 59 Department of Laboratory Medicine and Pathology, Mayo Clinic, Rochester, MN, United States of America; 60 Department of Health Sciences Research, Mayo Clinic, Rochester, MN, United States of America; 61 Cancer Epidemiology Centre, Cancer Council Victoria, Melbourne, Australia; 62 University of Hawaii Cancer Center, Honolulu, HI, United States of America; 63 Department of Medicine, McGill University, Montreal, Canada; 64 Division of Clinical Epidemiology, Royal Victoria Hospital, McGill University, Montreal, Canada; 65 Cancer Research Initiatives Foundation, Subang Jaya, Selangor, Malaysia; 66 Breast Cancer Research Unit, Cancer Research Institute, University Malaya Medical Centre, KualaLumpur, Malaysia; 67 Department of Genetics, Institute for Cancer Research, Radiumhospitalet, Oslo University Hospital, Oslo University Hospital, Oslo, Norway; 68 Institute of Clinical Medicine, Faculty of Medicine, University of Oslo, Oslo, Norway; 69 Department of Clinical Molecular Biology, Oslo University Hospital, University of Oslo, Oslo, Norway; 70 Division of Epidemiology, Department of Medicine, Vanderbilt-Ingram Cancer Center, Vanderbilt University School of Medicine, Nashville, TN, United States of America; 71 Laboratory of Cancer Genetics and Tumor Biology, Department of Clinical Chemistry and Biocenter Oulu, University of Oulu, Oulu, Finland; 72 Laboratory of Cancer Genetics and Tumor Biology, Northern Finland Laboratory Centre NordLab, Oulu, Finland; 73 Department of Oncology, Oulu University Hospital, University of Oulu, Oulu, Finland; 74 Lunenfeld-Tanenbaum Research Institute of Mount Sinai Hospital, Toronto, Canada; 75 Department of Molecular Genetics, University of Toronto, Toronto, Canada; 76 Prosserman Centre for Health Research, Lunenfeld-Tanenbaum Research Institute of Mount Sinai Hospital, Toronto, Canada; 77 Division of Epidemiology, Dalla Lana School of Public Health, University of Toronto, Toronto, Canada; 78 Department of Pathology, Leiden University Medical Center, Leiden, The Netherlands; 79 Department of Human Genetics, Leiden University Medical Center, Leiden, The Netherlands; 80 Department of Medical Oncology, Family Cancer Clinic, Erasmus MC Cancer Institute, Rotterdam, The Netherlands; 81 Division of Genetics and Epidemiology, The Institute of Cancer Research, London, UK; 82 Department of Medical Epidemiology and Biostatistics, Karolinska Institutet, Stockholm, Sweden; 83 Shanghai Center for Disease Control and Prevention, Shanghai, China; 84 Sheffield Cancer Research, Department of Oncology, University of Sheffield, Sheffield, UK; 85 Academic Unit of Pathology, Department of Neuroscience, University of Sheffield, Sheffield, UK; 86 International Epidemiology Institute, Rockville, MD, United States of America; 87 Centre for Cancer Genetic Epidemiology, Department of Oncology, University of Cambridge, Cambridge, UK; 88 Department of Preventive Medicine, Seoul National University College of Medicine, Seoul, Korea; 89 Department of Biomedical Sciences, Seoul National University College of Medicine, Seoul, Korea; 90 Cancer Research Institute, Seoul National University, Seoul, Korea; 91 Saw Swee Hock School of Public Health, National University of Singapore, Singapore, Singapore; 92 Department of Surgery, National University Health System, Singapore, Singapore; 93 Institute of Human Genetics, Pontificia Universidad Javeriana, Bogota, Colombia; 94 Department of Genetics and Pathology, Pomeranian Medical University, Szczecin, Poland; 95 National Cancer Institute, Bangkok, Thailand; 96 International Agency for Research on Cancer, Lyon, France; 97 Molecular Diagnostics Laboratory, IRRP, National Centre for Scientific Research "Demokritos", Athens, Greece; 98 School of Public Health, China Medical University, Taichung, Taiwan; 99 Taiwan Biobank, Institute of Biomedical Sciences, Academia Sinica, Taipei, Taiwan; 100 Cancer Center and Department of Surgery, Chung-Ho Memorial Hospital, Kaohsiung Medical University, Kaohsiung, Taiwan; 101 Centre Hospitalier Universitaire de Québec Research Center, Laval University, Québec City, Canada; 102 Usher Institute of Population Health Sciences and Informatics, University of Edinburgh, Edinburgh, UK; 103 Edinburgh Cancer Research UK Centre, University of Edinburgh, Edinburgh, UK; Hospital Authority, CHINA

## Abstract

The Cancer Genetic Markers of Susceptibility genome-wide association study (GWAS) originally identified a single nucleotide polymorphism (SNP) rs11249433 at 1p11.2 associated with breast cancer risk. To fine-map this locus, we genotyped 92 SNPs in a 900kb region (120,505,799–121,481,132) flanking rs11249433 in 45,276 breast cancer cases and 48,998 controls of European, Asian and African ancestry from 50 studies in the Breast Cancer Association Consortium. Genotyping was done using iCOGS, a custom-built array. Due to the complicated nature of the region on chr1p11.2: 120,300,000–120,505,798, that lies near the centromere and contains seven duplicated genomic segments, we restricted analyses to 429 SNPs excluding the duplicated regions (42 genotyped and 387 imputed). Per-allelic associations with breast cancer risk were estimated using logistic regression models adjusting for study and ancestry-specific principal components. The strongest association observed was with the original identified index SNP rs11249433 (minor allele frequency (MAF) 0.402; per-allele odds ratio (OR) = 1.10, 95% confidence interval (CI) 1.08–1.13, *P* = 1.49 x 10^-21^). The association for rs11249433 was limited to ER-positive breast cancers (test for heterogeneity *P*≤8.41 x 10^-5^). Additional analyses by other tumor characteristics showed stronger associations with moderately/well differentiated tumors and tumors of lobular histology. Although no significant eQTL associations were observed, in silico analyses showed that rs11249433 was located in a region that is likely a weak enhancer/promoter. Fine-mapping analysis of the 1p11.2 breast cancer susceptibility locus confirms this region to be limited to risk to cancers that are ER-positive.

## Introduction

Genome-wide association studies (GWAS) have identified over 90 common genetic variants associated with breast cancer risk [1–19]. A multi-stage GWAS, the Cancer Genetic Markers of Susceptibility (CGEMS) initiative, identified a single nucleotide polymorphism (SNP), rs11249433, associated with breast cancer risk. This SNP is located in the peri-centromeric region of chromosome 1p11.2, upstream of the *NOTCH2* and *FCGR1B* genes [12]. Further independent analysis confirmed this region as a breast cancer susceptibility locus associated with estrogen receptor (ER) positive but not ER-negative breast cancers [20–22], and more strongly associated with invasive lobular breast cancers than invasive ducal cancers [23]. Two independent meta-analyses on the basis of 15 case-control studies provided data supporting a significant association between rs11249433 and breast cancer among Caucasian populations but did not identify any significant association in Asian and African populations [24, 25].

Fine-scale mapping of the susceptibility regions identified by GWAS has the potential to further narrow down the relevant area of interest, identifying additional risk SNPs, and predicting potential functional mechanisms. Fine-mapping of the 1p11.2 locus among Chinese women (878 cases and 900 controls) identified a novel SNP rs2580520 as a variant significantly associated with breast cancer risk, which was not identified in European women [26]. However, fine-mapping has not been performed at this locus in a large population of multi-ethnic women. The Collaborative Oncological Gene-environment Study (COGS) designed and executed a collaborative genotyping and fine-mapping effort utilizing a custom built iSelect genotyping array (iCOGS) [8]. In this study we fine-mapped the1p11.2 breast cancer susceptibility locus utilizing the data generated through iCOGS, using both genotyped and imputed SNPs from over 50 case-control studies within the Breast Cancer Association Consortium (BCAC). Further, we determined whether the associated SNPs displayed heterogeneity by tumor subtype defined by ER-expression, as well as tumor grade and histology.

## Materials and Methods

### Study populations

Fifty breast cancer studies participating in the Breast Cancer Association Consortium (BCAC) were included in this analysis. The majority of the included studies were population-based or hospital-based case-control studies that included participants of European ancestry (41 studies), Asian ancestry (9 studies), and African ancestry (2 studies), totaling 45,276 breast cancer cases and 48,998 controls. Study participants were recruited under protocols approved by the Institutional Review Board at each institution, and all subjects provided written informed consent, as previously described [8]. For a list of all approving Institutional Review Boards by study, refer to Table A in S1 File.

### SNP selection, genotyping and imputation

Genotyping and quality control (QC) measures used in COGs have been described elsewhere [8]. In brief, excluded were SNPs with call rates of < 95%, with Hardy-Weinberg equilibrium deviation in controls at *P* < 1 x 10^-7^ and those with more than 2% of discrepant genotypes in duplicate samples across all COGS consortia. The 900 Kb genomic region for fine-mapping of the 1p11.2 locus (chr1p11.2: 120,300,000–121,185,600; based on build hg19) included all known SNPs correlated (*r*^2^> 0.1) with the index variant rs11249433. In total, 92 genotyped SNPs from the iCOGS array satisfied the initial QC metrics above.

We used imputation in order to estimate the genotypes at variants in the region not typed on the iCOGS array. Imputation was performed using IMPUTE2 [27], separately for each ethnic group. The IMPUTE2-info score and posterior probabilities at each SNP were used to evaluate imputation performance; scores ranged from 1 (high confidence) to 0 (no confidence). Markers with an IMPUTE2-info score < 0.9 or minor allele frequency (MAF) ≤ 3% were excluded from the association analyses as unreliable. Imputed genotypes where the maximum probability was <0.9 were considered unknown. For reference panels we used the International HapMap Project Phase 3 CEU data [28] and the 1000 Genome Project June 2010 release [29]. Based on these reference panels, genotypes at an additional 4,279 SNPs were reliably imputed across the 1p11.2 region.

After reviewing the 1p11.2 genomic region using the UCSC genome browser, we observed that there were several SNPs which mapped to multiple genomic regions due to duplication of genomic segments on both sides of the centromere on chromosome 1 [30, 31]. Therefore, we restricted our analysis to the region of 1p11.2 that excluded the following duplicated genomic segments: chr1:120531871–120697156; chr1:120747157–120936695; chr1:121086699–121133098; chr1:121160483–121222841; chr1:121280229–121351595; chr1:121361172–121418375 and chr1:121418377–121472478. The final analysis was therefore based on 42 genotyped and 387 imputed SNPs across ~210kb of genomic sequence. We also investigated data for a previously described SNP noted to be associated with risk in Asian populations, rs2580520 [26]. Unfortunately, the rs2580520 SNP was not genotyped in the iCOGS effort, is not curated in the 1000 Genomes database that was used for imputation of the 1p11.2 region (www.1000genomes.org/data [32]) and falls within the duplicated region noted above.

### Statistical analysis

The LD structure based on the 1000 Genomes CEU data was visualized using the R package snp.plotter [33]. A line graph was constructed displaying likelihood ratio statistics for recombination hotspot using SequenceLDhot software based on the background recombination rates inferred by PHASE v2.1. Physical locations of SNPs were based on hg19 and gene annotation and the LD plot was based on the NCBI RefSeq genes from the UCSC Genome Browser [34].

Standard logistic regression models were used where the common allele was the referent to assess the association of all genotyped and imputed SNPs with breast cancer risk and all analyses (overall and for breast cancer subtypes) used a 1-degree of freedom test (additive model) to estimate the per-allele odds ratio (OR) for the variant allele and corresponding 95% confidence interval (CI) for each SNP. Association analyses were adjusted for study and eight eigenvectors to capture population structure, obtained from principal component analyses [8]. P-values for trend from the Wald test are reported, imputed SNPs were handled using estimated allele dose. To identify SNPs independently associated with breast cancer risk within the 1p11.2 locus we conducted forward stepwise logistic regression analysis separately for each ethnicity (European, Asian and African) conditioning on rs11249433, the top SNP originally identified in CGEMS SNP and the top SNP at this locus in iCOGS. After identifying a novel independent signal, stepwise logistic regression analyses were repeated conditioning on the newly identified SNP rs146784183. Bonferonni adjusted significance was set at *P* < 7x10^-5^, corrected for 4,371 SNPs.

To determine if there were differences in the associated effects of the independent signals on different subtypes of breast cancer among women of European ancestry, we conducted stratified analyses according to subtypes defined by: 1) tumor histology (ductal/mixed, lobular, other), 2) tumor grade (well-differentiated, moderately-differentiated, poorly-differentiated), and 3) ER status (ER-positive or ER-negative) subtypes. To determine if SNP associations varied significantly between defined subtypes of breast cancer, we performed polytomous logistic regression models, and P-values for heterogeneity were obtained from case-case analysis for tumor subtypes (ER, tumor grade and tumor histology). Meta-analyses were performed using the random effects model to estimate the I^2^ statistic and p-value for heterogeneity by study.

### In silico functional analysis and eQTL data

To evaluate any possible functional implications of our top-associated SNPs, we assessed *in silico* functional data and expression quantitative trait loci (eQTL). Utilizing the UCSC Genome Browser and HaploReg v3 we reviewed ENCODE data to determine potentially altered regulatory motifs. RegulomeDB v1.1 was used to query publicly available eQTL data from multiple cell types associated with the identified SNPs and select SNPs significantly correlated to the tag SNP rs11249433.

## Results and Discussion

### Fine-scale mapping of the 1p11.2 locus

**Fig 1:**
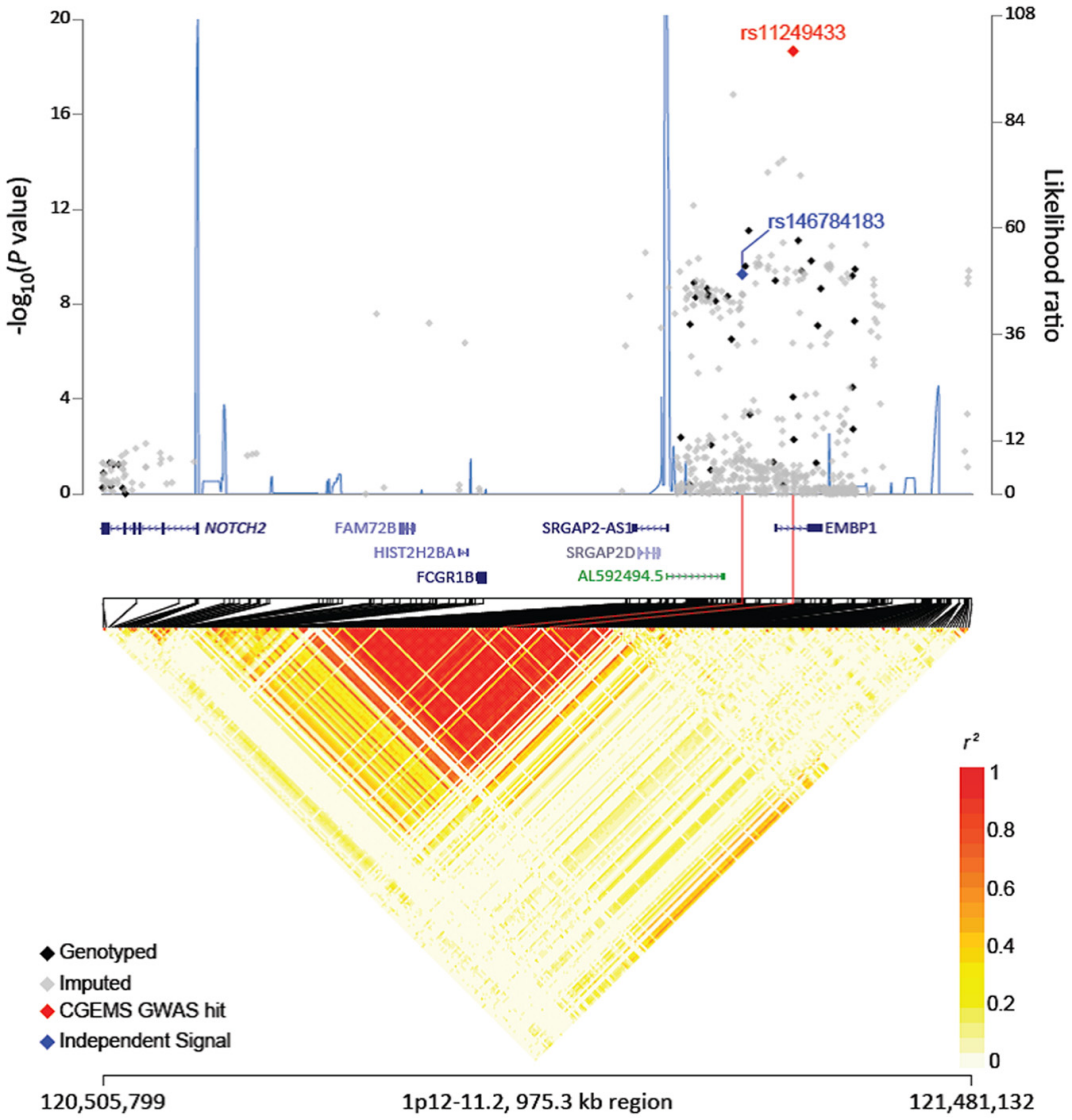
Regional plots of breast cancer association in 1p12-11.2. Regional plot of association result, recombination hotspots and linkage disequilibrium for the 1p12-11.2:120,505,799–121,481,132 breast cancer susceptibility loci. Association result from a trend test in—log10*P*values (y axis, left; red diamond, the top ranked breast cancer associated locus in the region; blue diamond, best conditioned analysis results conditioned on rs11249433; black diamonds, genotyped SNPs; gray diamonds, imputed SNPs) of the SNPs are shown according to their chromosomal positions (x axis). Linkage disequilibrium structure based on the 1000 Genomes CEU data (n = 85) was visualized by snp.plotter software. The line graph shows likelihood ratio statistics (y axis, right) for recombination hotspot by SequenceLDhot software based on the background recombination rates inferred by PHASE v2.1. Physical locations are based on hg19. Gene annotation was based on the NCBI RefSeq genes from the UCSC Genome Browser.

Following quality control and genomic restrictions, a total of 429 SNPs (42 genotyped and 387 imputed) were examined for their association with breast cancer risk. Fig 1 shows the genotyped and imputed SNPs analyzed in European women, plotted against corresponding chromosomal positions within 1p11.2. Gene annotations within this genomic region, including the *NOTCH2* gene, and the degree of linkage disequilibrium between the SNPs, are also shown in Fig 1.

### Breast cancer risk associations at the 1p11.2 locus

Of the 429 SNPs, 136 SNPs were associated with breast cancer risk overall in European women at P < 5x10^-8^ (Table B in S1 File and S1 Fig). The most significant association with breast cancer risk was observed for the previously identified rs11249433 SNP (MAF 0.402; per-G-allele OR = 1.10, 95% CI 1.08–1.13, P = 1.49 x 10^-21^, Table 1) [12]. To test for the existence of additional independent signals within the 1p11.2 locus, we conducted forward stepwise logistic regression analyses conditioning on the top SNP rs11249433. A second signal was identified corresponding to an imputed SNP rs146784183 (MAF 0.101; per-A-allele OR = 0.88, 95% CI 0.85–0.91, *P* = 1.27 x 10^-5^ after adjustment for rs11249433, Table 1). After adjustment for rs11249433, SNP rs146784183 was not strongly correlated with the index SNP (r^2^ = 0.086), and is located 57 kb telomeric from rs11249433, and closer to the *NOTCH2* gene. Stepwise regression analyses conditioning on both rs146784183 and rs11249433 did not result in the identification of any additional independent signals at this locus (Table C in S1 File). Meta-analyses demonstrated that results were similar across studies for association results seen for both rs11249433 (I^2^ = 0%, *P*-het_study_ = 0.844) and rs146784183 (I^2^ = 6.7%, *P*-het_study_ = 0.351).

**Table 1:** Two independent association signals at the 1p11.2 locus: Association results for breast cancer risk among women in BCAC, by ancestry.

Signal	SNP	Position^a^	Cases (N)	Controls (N)	Source^b^	Risk Allele	RAF^c^ Cases/Controls	*r*^2^^d^	OR (95% CI)^e^	*P*-trend
European Ancestry										
1	rs11249433	121280613	39,072	42,101	G	G	0.424/0.402	~	1.10 (1.08–1.13)	1.49E-21
									1.09 (1.06–1.11)^f^	6.54E-15
2	rs146784183	121223447	39,072	42,101	I	A	0.906/0.899	0.086	0.88 (0.85–0.91)	2.35E-12
									0.92 (0.88–0.95)^f^	1.27E-05
Asian Ancestry										
1	rs11249433	121280613	5,826	6,643	G	G	0.039/0.036	~	1.19 (1.04–1.36)	0.01
2	rs146784183	121223447	5,826	6,643	I	A	0.860/0.844	0.004	0.89 (0.82–0.96)	0.002

^a^Genomic coordinates are based on hg19.

^b^Source indicates whether the SNP was genotyped (G) or imputed (I) within the data used from the 1000 Genomes Project.

^c^Risk allele frequency (RAF) for cases/controls.

^d^Pair-wise linkage disequilibrium (r2) with the top SNP rs11249433 calculated using iCOGS (n = 84,396) data, for controls only.

^e^Per-allele odds ratios (OR) and 95% confidence intervals (95% CI) were estimated from logistic regression adjusted for study site and 7 principal components in Europeans and 2 principal components in women with Asian ancestry. The common allele was the referent for calculating odds ratio; the G-allele for both rs11249433 and rs146784183.

^f^Odds ratios (OR) and 95% confidence intervals (95% CI) were estimated from logistic regression mutually adjusted for rs146784183 and top SNP (rs11249433) along with study site and 7 principal components.

### Association analysis by estrogen receptor status in European women

We next determined whether risk associations at the 1p11.2 locus varied by estrogen receptor (ER) status; associations observed were limited to ER-positive (rs11249433: per-G-allele OR = 1.12, 95% CI 1.10–1.15, *P*-het = 9.88 x 10^-9^; rs146784183: per-A-allele OR = 0.86, 95% CI 0.82–0.89, *P-het* = 8.41 x 10^-5^; Table 2 and Table D in S1 File). Associations for these two SNPs among ER-negative breast cancers were null (rs11249433: per-G-allele OR = 1.00, 95% CI 0.95–1.05, *P* = 0.90; rs146784183: per-A-allele OR = 0.99, 95% CI 0.92–1.06, *P* = 0.68; Table 2 and Table D in S1 File). Meta-analyses stratified by estrogen receptor status demonstrated that results were similar across studies for association results seen for both rs11249433 (ER-positive: I^2^ = 0%, *P*-het_study_ = 0.846) and rs146784183 (ER-positive: I^2^ = 0%, *P*-het_study_ = 0.524).

**Table 2:** Two independent association signals at the 1p11.2 locus: Association results for breast cancer risk among European women in BCAC, by tumor characteristic.

Tumor Characteristic	Cases (N)	Signal	SNP	OR (95% CI)^a^	*P*-trend	*P*- heterogeneity^b^
**ER Status**						
ER-positive	6,315	1	rs11249433	1.12 (1.10–1.15)	4.09E-23	
ER-negative	21,610			1.00 (0.95–1.05)	0.90	9.88E-09
ER-positive	6,315	2	rs146784183	0.86 (0.82–0.89)	1.48E-12	
ER-negative	21,610			0.99 (0.92–1.06)	0.68	8.41E-05
**Tumor Grade**						
Well-differentiated	5,917	1	rs11249433	1.18 (1.14–1.23)	5.63E-17	
Moderately-differentiated	13,561			1.13 (1.10–1.16)	3.88E-17	
Poorly-differentiated	8,784			1.02 (0.98–1.05)	0.27	8.90E-11
Well-differentiated	5,917	2	rs146784183	0.84 (0.77–0.90)	3.23E-06	
Moderately-differentiated	13,561			0.85 (0.81–0.90)	6.45E-09	
Poorly-differentiated	8,784			0.96 (0.90–1.02)	0.20	8.80E-04
**Histology**						
Ductal/Mixed	22,308	1	rs11249433	1.08 (1.05–1.10)	5.07E-09	
Lobular	3,747			1.28 (1.22–1.35)	1.15E-23	
Other	2,563			1.12 (1.05–1.19)	0.0002	7.60E-11
Ductal/Mixed	22,308	2	rs146784183	0.90 (0.86–0.94)	1.69E-06	
Lobular	3,747			0.81 (0.74–0.89)	8.07E-06	
Other	2,563			0.90 (0.81–1.00)	0.05	0.11

^a^Odds ratios (OR) and 95% confidence intervals (95% CI) were estimated from logistic regression adjusted for study site and 7 principal components. The common allele was the referent for calculating odds ratio; the G-allele for both rs11249433 and rs146784183.

^b^P-heterogeneity tests whether SNP associations differ significantly by tumor characteristic.

### Association analysis by tumor grade and histology in European women

Assessment of risk associations by tumor grade showed that SNP rs11249433 was significantly associated with risk for well-differentiated tumors (per-G-allele OR = 1.18, 95% CI 1.14–1.23) and moderately-differentiated tumors (per-G-allele OR = 1.13, 95% CI 1.10–1.16), but not poorly-differentiated tumors (per-G-allele OR = 1.02, 95% CI 0.98–1.05; *P* -het = 8.90 x 10^-11^,Table 2 and Table E in S1 File). Similarly, SNP rs146784183 showed significant associations for well and moderately-differentiated tumors, but not poorly-differentiated tumors in (*P* -heterogeneity = 8.80 x 10^-4^,Table 2 and Table E in S1 File). Results were similar when assessing heterogeneity by tumor grade only among ER-positive breast cancers, there were no significant associations for poorly-differentiated ER-positive tumors (Table F in S1 File).

Differential risk associations for rs11249433 was also seen by tumor histology, where associations were strongest for lobular tumors (per-G-allele OR = 1.28, 95% CI 1.22–1.35; *P* -het = 7.60 x 10^-11^), and less so for ductal/mixed or other tumor histology (Table 2). Significant risk differences by tumor histology were not observed for SNP rs146784183 (*P* -heterogeneity = 0.11), though the risk reduction associated with this SNP was strongest for lobular tumors (Table 2). Of the 160 genotyped and imputed SNPs found to be significantly associated with lobular breast tumors at a Bonferroni adjusted *P* < 7 x 10^-5^, 127 (79%) were also associated with ductal/mixed tumors, and only 30 (19%) of those also associated with tumors of other histology (Table G in S1 File).

### Analysis of index SNPs in different ethnic groups

We also examined breast cancer risk associations among participants in the nine case-control studies that included women of Asian ancestry (Table 2, S1 Fig and Table H in S1 File). The degree of linkage disequilibrium between the SNPs in this region was examined using HapMap data (S2 Fig).

The top SNP among European women, rs11249433, was also associated with breast cancer risk among Asian women (per-G-allele OR = 1.19, 95% CI 1.04–1.36; *P* = 0.01, Table 1 and S1 Fig). Although this SNP is rare in this population (MAF = 0.037), the OR was consistent with that in Europeans. SNP rs146784183 was also associated with breast cancer risk among Asian women (per-A-allele OR = 0.89, 95% CI 0.82–0.96; *P* = 0.002, Table 1 and S1 Fig).

The most strongly associated SNP within the Asian population, genotyped SNP rs115775083, was found to be significantly associated with breast cancer risk overall within the Asian population (per-T-allele OR = 1.78, 95% CI 1.43–2.20, *P* = 1.52 x 10^-7^, Table H in S1 File). The rs115775083 genotyped SNP is a rare variant among Asian women with a MAF = 0.011. This genotype is also rare among European women (MAF = 0.016) but not associated with breast cancer risk (per-T-allele OR = 0.95, 95% CI 0.88–1.02, *P* = 0.15). SNP rs115775083 is not correlated with the rs11249433 and rs146784183 SNPs identified to be associated with breast cancer risk in European women (*r*^2^ < 0.01). Conditioning on the top SNP identified in the women of Asian did not identify any novel signals within the 1p11.2 locus, but did reaffirm SNP rs115775083 as the most significant signal among Asian women (Table I in S1 File). Similar analyses were performed among women with African Ancestry using data from two BCAC studies (N = 378 cases and N = 254 controls). There were no SNPs within the 1p11.2 locus found to be significantly associated with breast cancer risk after adjusting for multiple comparisons (Table J in S1 File and S1 Fig).

### In silico functional and eQTL data

SNP rs11249433, was strongly correlated with one other SNP, rs12134101 (r^2^ = 0.943), which showed a similar association with risk (both for overall and ER-positive breast cancer). All other SNPs were less strongly associated with risk (likelihood ratio < 1:1000 relative to rs11249433), suggesting that one or both SNPs rs11249433 and rs12134101 are likely to be causally implicated in breast cancer risk.

*In silico* analyses showed that SNP rs11249433 was found to be located within a weak enhancer and weak promoter in myoblasts and leukemia cells, respectively. Also, this SNP was located within a region of DNase I hypersensitivity and histone H3K27 acetylation in multiple cell types including T47D and MCF7 breast cancer cell lines. There were no proposed regulatory motifs altered by SNP rs146784183, and neither rs11249433 nor rs146784183 were found to have any significant eQTL associations.

In this large-scale fine-mapping analysis of nearly 50,000 breast cancer cases and 50,000 controls within the Breast Cancer Association Consortium (BCAC), we found index SNP rs11249433 to be the strongest signal within the 1p11.2 locus associated with breast cancer risk in European women. An additional association signal was identified, rs146784183, that was independent of the index SNP for overall breast cancer risk. Neither signal was found to be significantly associated with breast cancer risk among women with Asian or African ancestry, after adjusting for multiple comparisons. Notably, rs11249433 and rs146784183 displayed significant heterogeneity in risk associations by important tumor characteristics including ER status, tumor grade and histology. Our findings highlight the value of fine-mapping analyses to identify novel risk associations, and the utility of performing large-scale genotyping projects within varied ethnic populations to aid in narrowing down the genomic area relevant to future functional analyses.

Fine-mapping the 1p11.2 locus was complex due to the proximity to the centromere and the presence of duplicate genomic segments. As such, we employed strict quality control measures to increase our likelihood for finding true association signals. In this study we have identified SNP rs146784183 as a novel independent signal within the 1p11.2 locus among European women. SNP rs146784183 and the index SNP were not correlated (*r*^2^ = 0.086), and this newly identified SNP is located about 57 kb telomeric from the index SNP, and closer to the *NOTCH2* gene.

Our findings concur with previous research identifying rs11249433 as a SNP displaying heterogeneity by important tumor characteristics including ER status and histology. Specifically, rs11249433 was found to be more strongly associated with tumors of lobular histology and those that were ER-positive [20–22]. Further, we have recently shown that this SNP was more strongly associated with tumors having low E-cadherin breast tissue expression compared to E-cadherin high tumors [22]. Our current and previous findings for SNP rs11249433 are consistent given that expression of the E-cadherin tumor suppresor protein is frequently lost in tumors of lobular histology.

We did not identify any eQTL signals for either rs11249433 or rs146784183. *In silico* analyses showed that rs11249433 is situated in a DNase I hypersensitive region which contains open chromatin with histone marks, suggestive that this region might be a weak enhancer in some cell types [35]. SNP rs11249433 is located upstream of the *NOTCH2* gene on chromosome 1. The NOTCH signaling pathway has been frequently implicated in breast cancer development though the exact function of *NOTCH2* in this process is not well characterized [36–40]. Interestingly, the *NOTCH2* gene was shown to be associated with super-enhancers, or large clusters of transcriptional enhancers that drive expression of genes that function in the acquisition of hallmark capabilities in cancer [41]. Dysregulation of the NOTCH signaling pathway has been implicated in breast cancer initiation and progression; this pathway is also considered as the target for novel therapeutics [36–40]. Consequently, though rs11249433 is located within a weak enhancer, it is plausible that it participates in transcriptional regulation through the function of a larger super-enhancer that contributes to tumor pathology.

In the current study we did not perform functional analyses, however, in a study of 180 breast tumors Fu and colleagues found that carriers of the risk genotypes of rs11249433 (AG/GG) were associated with increased mRNA expression of the *NOTCH2* gene [20–22]. Further, expression of *NOTCH2* was highest in ER-positive/TP53 wild-type tumors. This study supports the potential regulation of *NOTCH2* gene expression by SNP rs11249433 and in turn, is in line with our observation that this SNP is specific to ER-positive breast tumors. In a separate study of NOTCH2 protein expression in breast cancer, NOTCH2 levels were found to be high in well-differentiated tumors and low in poorly-differentiated tumors [42]. If, as suggested by Fu et al. [21], rs11249433 contributes to the increased expression of *NOTCH2*, the observation by Parr and colleagues that NOTCH2 is highest among well-differentiated tumors, supports our findings for low grade, well-differentiated tumors. However, without direct experimental evidence, it is difficult to determine the functional implications of these SNPs with certainty. While it is possible that these two variants (rs11249433 and rs146784183) are influencing different genes, however, the patterns of association with breast cancer sub-types suggest that they may affect similar biological and/or signaling processes.

Our analyses in a diverse population of women showed that the top association signals found in European women showed similar associations in women of Asian ancestry, although associations were weaker. However, no significant signals were observed among women with African ancestry. These findings support what has been previously shown in multi-ethnic studies of the 1p11.2 locus [24, 25, 43]. Among Asian women, a rare variant, SNP rs115775083 was found to be the strongest association signal for breast cancer overall. This region of chromosome 1 and its association with breast cancer has been examined among Chinese women. Jiang and colleagues assessed the association of seven tagging SNPs, including rs11249433, within a 277 kb region of 1p11.2 [26]. In the Jiang study, the authors observed borderline significant associations of rs11249433 with breast cancer risk in their population of Chinese women. However, given that this SNP is rare among women with Asian ancestry, the absence of a significant association is likely due to decreased power caused by insufficient numbers of cases harboring the risk allele. rs115775083, the top SNP among Asian women in our population, was not included among the seven SNPs assessed in the Jiang study [26]. We were unable to duplicate the findings from Jiang et al. [26] which identified rs2580520 as a significant association signal among Chinese women. The rs2580520 SNP was not genotyped as part of the iCOGs effort, is not found in the 1000 Genomes Project Phase 1 data [32] which was used for imputation, and maps to a suspected duplicated region. These data illustrate the challenges of genotyping this complex region. Though no significant signals were found among women with African Ancestry, examining the regional plots among European, Asian and African women, association analysis suggests that the relevant area of interest for future studies lies within the interval spanning chr1p11.2: 121,105,799–121,405,799.

The strengths of our study are in analysis of a very large data set, which includes subjects of European, Asian and African ancestry; and availability of detailed genetic and tumor pathology data, which allowed us to refine these risk associations by pathologic subtypes of breast cancer. Moreover, the findings observed in this pooled analysis did not differ significantly by study. Our study was limited by the available genomic information of the 1p11.2 region. However, the genomic map of the peri-centromeric region that harbors our region of interest was significantly improved in the latest build of the reference human genome. Due to this improvement, some genomic gaps were filled and some new pseudogene transcripts were mapped in the region; this could potentially increase SNP coverage and improve fine-mapping quality.

## Conclusions

In summary, we showed the 1p11.2 locus is specific for ER-positive breast cancers and provided data to narrow the relevant area of interests for future functional studies, which should provide further insights into the underlying causal SNPs responsible for its association with breast cancer.

## Supporting Information

S1 Fig Regional plots of 1p12-11.2 breast cancer associations in women of European, Asian and African Ancestry. Regional plot of association results for the 1p12-11.2:120,505,799–121,481,132 breast cancer susceptibility loci from women of European (top panel), Asian (middle panel) and African (lower panel) ancestry. Association result from a trend test in—log10*P*values (y axis, left; red diamond, the top ranked breast cancer associated locus among European women; blue diamond, best conditioned analysis results conditioned on rs11249433 among European women) of the SNPs are shown according to their chromosomal positions (x axis). Physical locations are based on hg19.
(DOC)

S2 Fig LD plots for CEU, Asians and Yoruba (YRI) based on HapMAP version 3. Linkage disequilibrium (LD) plots for (A) women with ancestry from northern and western Europe (CEU), (B) Asian ancestry and (C) Yoruba (YRI) women with West African ancestry based on HapMAP version 3, chromosome 1: 120505–121481 kb. Index SNP rs11249433 is circled in red.
(DOCX)

S1 File Table A: List of studies and ethics approvals. Table B: Genotyped and imputed SNPs at the 1p11.2 locus associated with overall breast cancer risk at genome-wide significance (p < 5x10-8) among European women in BCAC. Table C: Genotyped and imputed SNPs at the 1p11.2 locus associated breast cancer risk at genome-wide significance (p < 5x10-8) after conditioning on SNP rs146784183 among European women in BCAC. Table D: Genotyped and imputed SNPs at the 1p11.2 locus associated with ER-positive breast cancer risk at Bonferroni adjusted significance (p < 7x10-5) and the corresponding association with ER-negative breast cancer risk among European women in BCAC. Table E: Genotyped and imputed SNPs at the 1p11.2 locus associated with well differentiated breast cancer risk at Bonferroni adjusted significance (p < 7x10-5) and the corresponding association with moderately differentiated and poorly differentiated breast cancer risk among European women in BCAC. Table F: Two independent association signals at the 1p11.2 locus: Association results for breast cancer risk among European women in BCAC, by tumor characteristic. Table G: Genotyped and imputed SNPs at the 1p11.2 locus associated with lobular breast cancer risk at Bonferroni adjusted significance (p < 7x10-5) and the corresponding association with ductal or mixed and other breast cancer risk among European women in BCAC. Table H: Top 5 SNPs at the 1p11.2 locus and their association with breast cancer risk among women with Asian ancestry in BCAC. Table I: Results of association analyses after conditioning on the top SNP (rs115775083) identified among women with Asian Ancestry. Table J: Two independent association signals at the 1p11.2 locus, lack of association with breast cancer risk among women with African Ancestry. Table K: Acknowledgements and funding.
(XLSX)
