# Supplementary material for: Fine-Mapping of the 1p11.2 Breast Cancer Susceptibility Locus
Source: PLoS One. 2016 Aug 24;11(8):e0160316. doi: 10.1371/journal.pone.0160316 (PMC4996485; doi:10.1371/journal.pone.0160316)
Supplement: S1 Fig — Regional plot of association results for the 1p12-11.2:120,505,799–121,481,132 breast cancer susceptibility loci from women of European (top panel), Asian (middle panel) and African (lower panel) ancestry. Association result from a trend test in—log10Pvalues (y axis, left; red diamond, the top ranked breast cancer associated locus among European women; blue diamond, best conditioned analysis results conditioned on rs11249433 among European women) of the SNPs are shown according to their chromosomal positions (x axis). Physical locations are based on hg19. (DOC) [file pone.0160316.s001.doc]

**S1 Fig 1. Regional plots of 1p12-11.2 breast cancer associations in women of European, Asian and African Ancestry.**

**
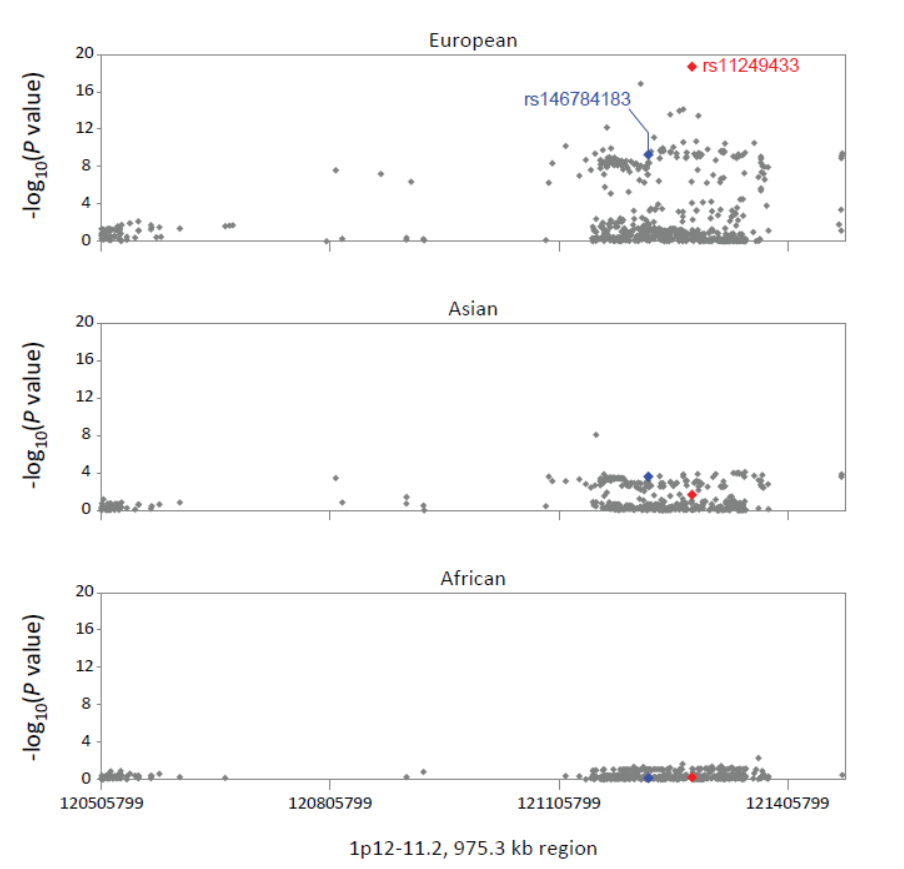
**
