# Supplementary figures and images for: Fine-Mapping of the 1p11.2 Breast Cancer Susceptibility Locus
Source: PLoS One. 2016 Aug 24;11(8):e0160316. doi: 10.1371/journal.pone.0160316 (PMC4996485; doi:10.1371/journal.pone.0160316)

**S2 Fig.** **LD plots for CEU, Asians and Yoruba (YRI) based on HapMAP version 3.**


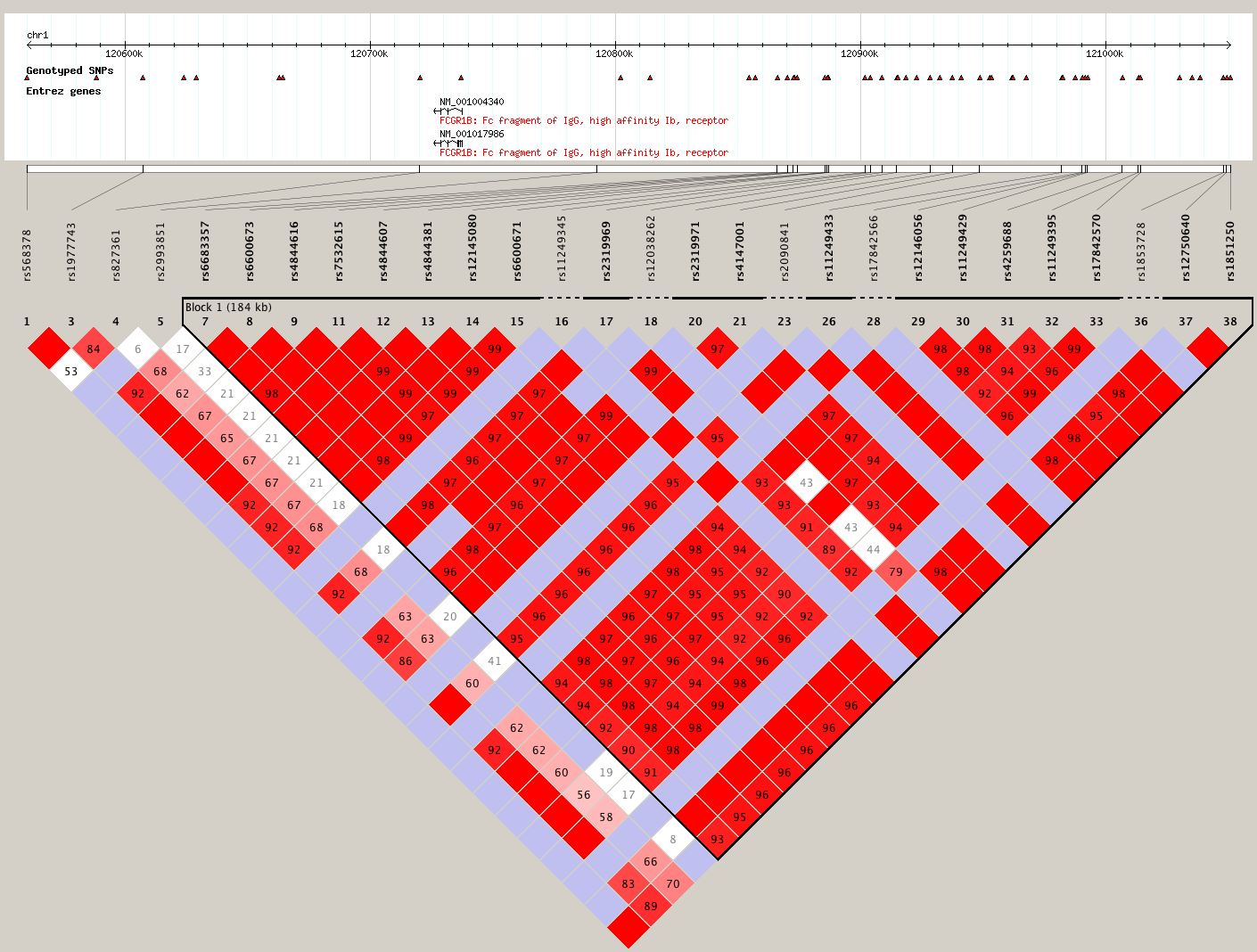


CEU


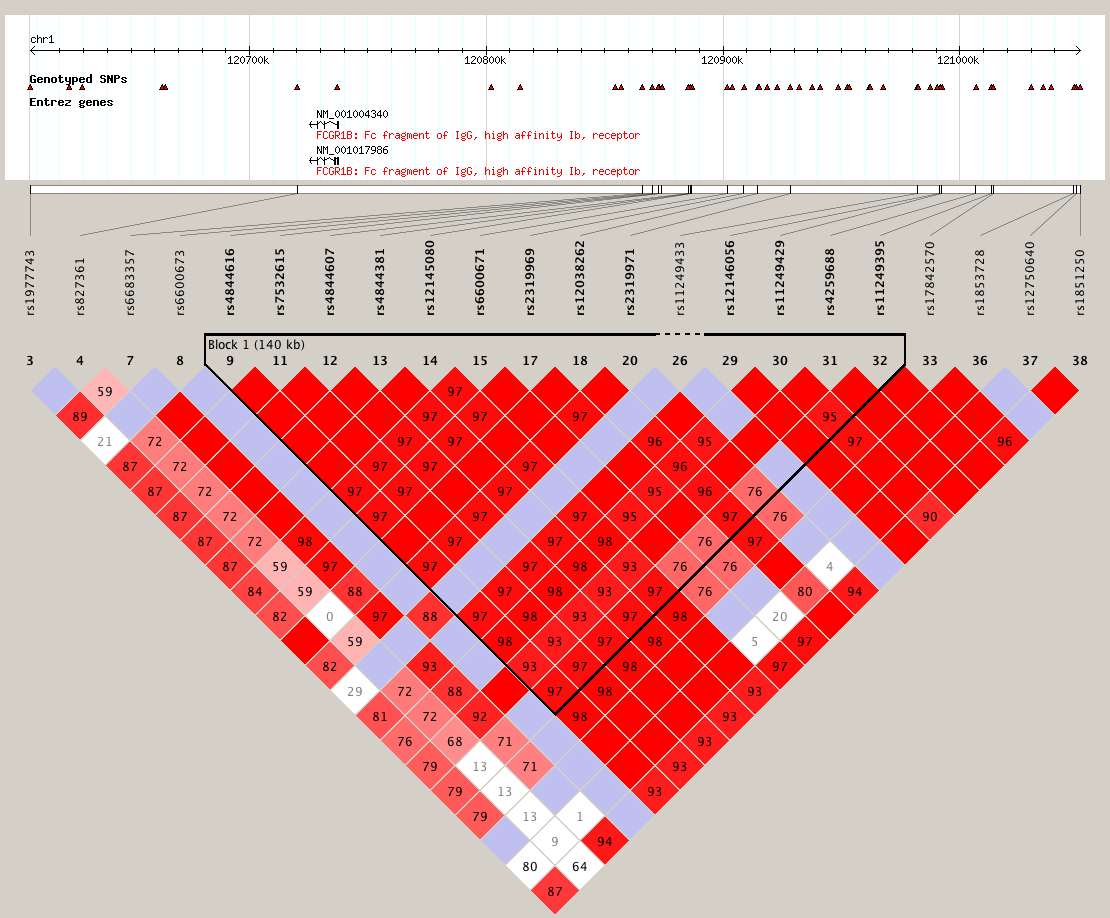


Asians

YRI


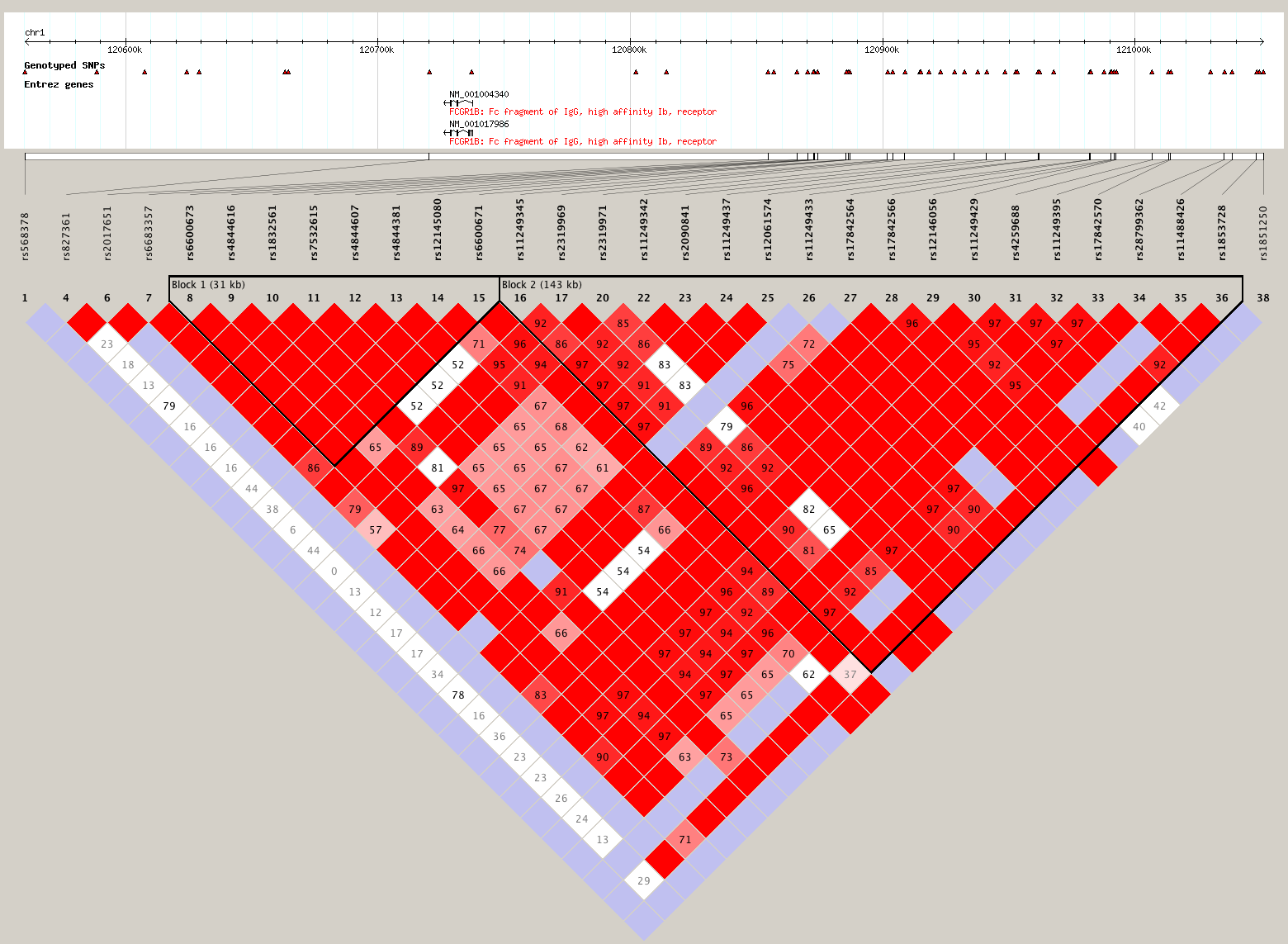

Supplement: S2 Fig — Linkage disequilibrium (LD) plots for (A) women with ancestry from northern and western Europe (CEU), (B) Asian ancestry and (C) Yoruba (YRI) women with West African ancestry based on HapMAP version 3, chromosome 1: 120505–121481 kb. Index SNP rs11249433 is circled in red. (DOCX) [file pone.0160316.s002.docx]
